# Neutral polysaccharide from *Panaxnotoginseng* enhanced cyclophosphamide antitumor efficacy in hepatoma H22-bearing mice

**DOI:** 10.1186/s12885-020-07742-z

**Published:** 2021-01-07

**Authors:** Yan-Hong Liu, Hua-Yan Qin, Yuan-Yuan Zhong, Shuang Li, Hua-Jing Wang, Hong Wang, Li-Ling Chen, Xiang Tang, Ya-Lin Li, Zhong-Yi Qian, Huai-Yu Li, Lei Zhang, Tong Chen

**Affiliations:** 1 School of Pharmaceutical Sciences and Yunnan Key Laboratory of Pharmacology for Natural Products, Kunming Medical University, Kunming, 650500 China; 2 Key Laboratory of Medicinal Chemistry for Natural Resource, Ministry of Education, Yunnan University, Kunming, 650091 China; 3 Department of Geriatrics, the First Affiliated Hospital of Kunming Medical University, Kunming, 650032 China; 4 Clinical skills center, Kunming Medical University, Kunming, 650223 China; 5 Basic Medical College of Kunming Medical University, Kunming, 650500 China; 6 Dali Nursing Vocational College, Dali, 671006 China; 7 Yunnan University of Traditional Chinese Medicine, Kunming, 650200 China

**Keywords:** *Panax notoginseng*, Neutral polysaccharide from *Panax notoginseng*, Purification, Myelosuppression, Immunosuppression, Combined medication, Liver cancer

## Abstract

**Background:**

Our previous studies demonstrated that the administration of crude Polysaccharide from *Panax notoginseng* (CPPN) can effectively prolong the lifespan of tumor-bearing mice via boosting the host immune system as well as weak cytotoxicity against hepatocellular carcinoma (HCC). In the present study, Neutral Polysaccharide (NPPN) were further purified from crude polysaccharide isolated from *panax notoginseng*. The effects of NPPN on the immune function and hematopoietic function of mice with low immunity and myelosuppression induced by cyclophosphamide (CTX) were investigated. The effect of NPPN combined with CTX on the tumor inhibition rate of the H22 tumor-bearing mice and the impact of NPPN on the proliferation of H22 liver cancer cells in vitro were investigated.

**Methods:**

CPPN was obtained by water extraction and alcohol precipitation method, and further purified by DEAE Sepharose Fast Flow ion exchange resin column. NPPN was added to the immunosuppressed with myelosuppression mice induced by CTX. Thymus index, spleen index, lymphocyte proliferation stimulation index by adding of concanavalin A, determination of serum hemolysin, NK cell activity assay, mice carbon clearance experiment, blood count tests were detected. The tumor inhibition rate of the H22 tumor-bearing mice treated with NPPN combined with CTX was recorded.

**Results:**

NPPN and 4 kinds of acid polysaccharide from *Panax notoginseng* (APPN) were successfully isolated from the CPPN by DEAE Sepharose Fast Flow ion exchange resin column. NPPN inhibited the growth of H22 cells and significantly increase the tumor inhibition rate of the H22 tumor-bearing mice combined with CTX. The elevation of the cellular and humoral immunity levels as well as a variety of blood count tests indicators of immunosuppressive with myelosuppression mice may contribute to the antitumor activity of NPPN.

**Conclusion:**

NPPN has a potential antitumor activity for the treatment of liver cancer combined with cyclophosphamide.

## Background

Primary liver cancer is one of the most common cancer in the world. Approximately 70 to 85% of primary liver cancer cases are hepatocellular carcinoma (HCC), which is the third leading cause of cancer-related death following gastric cancer and lung cancer, and causes approximately one million deaths every year worldwide [1]. The early stage of HCC usually shows no or only mild symptoms. HCC usually is diagnosed at the late stage when the chance of the curative treatments such as resection, transplantation, or local ablation has gone. As a result, the median survival following diagnosis is only approximately 6–20 months [1, 2]. The late stage HCC is commonly treated by systemic anticancer therapies such as chemotherapy with unsatisfied efficacy. In addition, chemotherapy such as Cyclophosphamide (CTX) massively depletes bone marrow progenitor cells resulting in anemia, neutropenia, and thrombocytopenia. Patients may suffer from complications such as fatigue, dizziness, bruising, hemorrhage, and potentially fatal opportunistic infections. To reduce these side effects, drug dosage and frequency may have to be limited. In turn, the treatment efficacy may be compromised [3–5] CTX is one of the most widely used antitumor agents in clinical chemotherapy for the treatment of a broad spectrum of human cancers including lymphomas, some forms of brain cancer, leukemia as well as some solid tumors. It belongs to the nitrogen mustard group which can induce DNA damages in the fast dividing tumor cells by nonspecifically alkylating the DNA of tumor cells. When the DNA damages are severe enough then tumor cells are forced to undergo apoptosis modulated by p53, a protein maintaining the genome integrity. Thus, tumor growth could be suppressed. However, serious side effects such as myelosuppression, one of the most important blood toxicities, are often caused by the application of CTX [6]. The post-chemotherapy leucopenia is the most common phenomenon and may easily result in a concurrent lethal infection. Currently, the most common treatments for chemotherapy-induced myelosuppression are blood transfusion, growth factor injections, or bone marrow transplantation for an inevitable complete myeloablation. Such managements are costly and limited effectiveness [7]. New approaches are urgently needed to ameliorate the immune system for the prevention of chemotherapy-induced bone marrow suppression and improvement of cancer patient survival and quality of life. It has been demonstrated that Polysaccharides from various sources enhanced antitumor therapy via boosting the immune system of cancer patients with relatively nontoxic [8–11]. Polysaccharide from *Panax notoginseng* (PPN) has been shown to stimulate murine spleen lymphocyte proliferation in vivo and in vitro. It also abolished the suppression of the T cell by cyclosporin A [11]. The 1500 k Da fraction of the extraction from roots of *Panax notoginseng* activated the reticuloendothelial system [12]*.* IFN-γ and IFN-α in mouse spleen lymphocytes and peritoneal macrophage cell culture have been eleven by the application of the water soluble, high molecular weight fraction of an extraction of *Panax notoginseng* [8]. In our previous studies, we successfully developed a reliable and effective method to extract alcohol insoluble CPPN from the industrial notoginsenosides extract residues with the contents of polysaccharides as 56% [13]. The administration of CPPN alone can effectively prolong the lifespan of tumor-bearing mice via boosting the host immune system as well as weak cytotoxicity against HCC [14].

In the present study, to validate the polysaccharides of CPPN playing a major role in immuno-stimulation, NPPN fraction has been further purified from CPPN to increase the content of polysaccharides from 56 to 88.4%. To evaluate the effects of NPPN on the immune function and hematopoietic function of Kunming mice, NPPN has been combined with CTX and applied to H22 tumor-bearing mice. Though CTX slowed down tumor growth, it resulted in sever immunosuppressive and myelosuppression in mice. The application of NPPN improved hematopoietic status while combined with CTX, consequentially, enhanced the antitumor efficacy of CTX.

## Methods

### Experimental animals

Female Kunming mice (18–22 g) were provided by the Laboratory Animal Center of Kunming Medical University. Animals were housed in an SPF environment. Environment conditions were a temperature of 20 ± 4 °C, the humidity of 50 ± 10%, the lighting of 350 lx, and a 12/12 light/dark cycle. During housing, animals were given a normal diet. All animals were monitored twice daily for health status, no abnormal reaction appeared in the all group. All groups of mice were put to death by cervical dislocation after inhalation of ether and coma. All experimental procedures were performed in accordance with the Guidelines of Animal Experiments from the Committee of Medical Ethics, National Health Department of China and approved by the Ethics Committee of Kunming Medical University. All sections of this report adhere to the ARRIVE Guidelines for reporting animal research. A completed ARRIVE Guidelines checklist is included in checklist S1.

### Extraction of CPPN

The extraction of CPPN from the root of *Panax notoginseng* has been described previously [14]. Briefly, 100 g dried *Panax notoginseng* medicinal residue was mixed with 1000 g distilled water in a 2000 mL beaker. The beaker was then kept in a boiling water bath for 6 h then taken out and cooled down to room temperature. The liquid was poured out as the first extraction. Then, 800 g distilled water was added to the remaining solid portion and the beaker was kept in a boiling water bath again for an additional 6 h. Then the liquid was collected as the second extraction. Followed, 600 g distilled water was added to the remaining solid portion. The beaker was kept in a boiling water bath again for an additional 6 h. Then, the liquid portion was collected as the third extraction. All three extractions were combined then centrifuged at 8000 rpm for 10 min. The supernatant was collected and condensed to 1/10 of the original volume in a vacuum condenser at 80 °C. Followed, three volumes of 100% ethanol were gradually added to the concentrated extraction with stirring. The mixture was then kept at 4 °C for 24 h followed by centrifugation at 8000 rpm for 10 min. After the removal of the supernatant, the CPPN was washed with 75% ethanol and dried in a drying oven at 50 °C.

### Extract NPPN from CPPN

NPPN was further extracted from CPPN by DEAE Sepharose Fast Flow anion exchange chromatography column (GE Healthcare, Little Chalfont, Buckinghamshire, UK) (24 × 400 mm) according to manufactory instructions. CPPN was dissolved in distilled water at a concentration of 30 mg·mL^− 1^. The solution was centrifuged at 8000 rpm for 10 min. The supernatant was then filtered through a 0.45 μm microporous membrane and 25 mL solution was loaded to the column. The first elution used distilled water and collected by 5 mL eluate per tube was collected. Until the anthrone-sulfuric acid reagent was added to the last tube and heat in a water bath for 15 min and cool in an ice bath for 15 min and no coloration was observed. Replace the eluent and elute sequentially with 0.1, 0.2, 0.3, and 0.4 mol·L^− 1^ sodium chloride solution, and the eluent was collected, 5 mL per tube, and collected until the anthrone-sulfuric acid reagent was added and heat in a water bath for 15 min and cool in an ice bath for 15 min and no coloration was observed. Determination of the polysaccharide content of *Panax notoginseng* by the method of anthrone-sulfuric acid [15]. The number of eluted tubes was plotted on the abscissa and the absorbance was plotted on the ordinate. The eluent was combined according to the elution curve. And then the eluent was concentrated under reduced pressure by rotary evaporator at 80 °C to about 50 mL. Transfer 50 mL of rotary evaporation liquid to a dialysis bag (Biosharp, Jiangsu, China).having a molecular weight of 14,000 Da by a pipette. The dialysis bag was sealed and placed in 2000 mL of distilled water for dialysis for 36 h and changed every 9 h.

### Determination of molecular weight by high performance gel permeation chromatography

The weight average molecular weight (Mw), number average molecular weight (Mn), and dispersity were measured by high performance gel permeation Chromatography (HPGPC). Chromatographic conditions: Agilent 1260 HPLC system (Agilent Technologies, Santa Clara, CA, USA); data processing system: Agilent GPC software. Mobile phase: 0.1 mol·L^− 1^ NaCl; column: Shodex Ohpak SB-804HQ gel column (Shodex, Japan); flow rate: 0.5 mL·min^− 1^; Column temperature: 35 °C; detector: differential detector; injection volume: 20 μL. A standard curve was established using Dextran T series standards (National Institutes for Food and Drug Control, Beijing, China) (standard molecular weights of 4600, 7100, 10,000, 21,400, 41,100, 84,400, 133,800 Da, respectively). The above-mentioned one part of NPPN and the four parts of APPN were dissolved into an aqueous solution having a concentration of about 10 mg·mL^− 1^, and filtered through a 0.22 μm microporous membrane, and then injected.

### Determination of the content of PPN by an anthrone-sulfuric acid method

Determination of the polysaccharide content of *Panax notoginseng* by the method of anthrone-sulfuric acid [15]. Preparation of an anthrone-sulfuric acid reagent: Take about 0.1 g of anthrone, accurately weighed, add 80% sulfuric acid solution to dissolve, transfer to a 100 mL volumetric flask, and place it in a dark place. Preparation of standards solution and drawing of its absorbance value and concentration standard curve: Take about 10 mg of D-anhydrous glucose reference substance (National Institutes for Food and Drug Control, Beijing, China) dried to constant weight at 105 °C, accurately weighed, placed in a 50 mL volumetric flask, plus dissolve the appropriate amount of distilled water, add distilled water to volume, and store at 4 °C. Precisely measure 0.0, 0.2, 0.4, 0.6, 0.8, 1.0 mL of the reference solution in a test tube, add distilled water to 2.0 mL, accurately measure 5.0 mL of the anthrone sulfuric acid reagent into the test tube, shake well, and heat in a boiling water bath for 15 mins, quickly removed into the ice water bath for 15 mins. The absorbance value of the reference solution at 625 nm was determined. Taking the absorbance of the D-anhydrous glucose reference solution as the ordinate and the concentration of the D-anhydrous glucose reference solution as the abscissa, a standard curve of the absorbance value and the concentration of the reference solution is plotted.

Determination of Polysaccharide content of *Panax notoginseng*: Take about 10 mg of the test sample, accurately weigh it into a beaker, add distilled water, dissolve in a water bath, transfer to a 25 mL volumetric flask and add distilled water to volume. Accurately measure 0.4 mL in a test tube, measure the absorbance value according to the above method, and take the absorbance value into the above standard curve to calculate the concentration of the test sample, and calculate the content of the polysaccharide in the test sample.

### Establishment and grouping of cyclophosphamide-induced immunosuppression and myelosuppression mouse models

SPF Kunming mice were randomly divided into 5 groups, 10 in each group, 5 groups were: low, medium, and high doses of NPPN groups (93 mg·kg^− 1^, 188 mg·kg^− 1^, 375 mg·kg^− 1^), model group, and normal group. The low, medium, and high doses of NPPN groups were continuously intragastrically administered with different concentrations of NPPN solution for 10 days. The model group and the normal group were continuously administrated with normal saline for 10 days. On the 6th day of intragastric administration, except for the normal group, the other groups were intraperitoneally injected with CTX (Shengdi, Jiangsu, China) (80 mg·kg^− 1^·d) for 5 days, and the indexes were determined on the 11th day.

### Determination of thymus and spleen index

On the 11th day, mice in each group were killed after weighed. Then, the thymus and spleen were removed and weighed. The thymus index was calculated as thymus weight divided by body weight. Spleen index was calculated as spleen weight divided by body weight.

### Determination of lymphocyte proliferation stimulation index by adding of concanavalin a

Preparation of mouse spleen lymphocytes [16]: The spleen of mice was isolated under aseptic conditions, placed in a dish containing an appropriate amount of sterile Hank’s solution (Solarbio, Beijing, China), and the fat and connective tissue were removed, ground in a mortar, and filtered through a 200 mesh screen. The filtrate was centrifuged at 1000 r·min^− 1^ for 10 mins, the supernatant was discarded, 3 volumes of red blood cell lysate (Solarbio, Beijing, China) was added and the cells were lysed for 10 mins on ice. After centrifugation at 4 °C, the supernatant was discarded, Hank’s solution washed the cells twice, and the supernatant was discarded after centrifugation at 4 °C. Resuspend in 1.5 mL of complete medium, count live cells, and adjust the number of cells to 2 × 10^6^ cells/mL. The mouse spleen cell suspension (2 × 10^6^ cells/mL) was inoculated into a 96-well culture plate at 200 μL/well. Each sample was provided with a stimulation well and control well, and three parallel holes were set for each. The stimulation well was added with Concanavalin A (Solarbio, Beijing, China) containing 0.1 mg·mL^− 1^ 15 μL of the culture solution. The cells were placed in a 37 °C 5% CO_2_ incubator for 44 h, and then 20 μL (5 mg·mL^− 1^) of the MTT solution (Solarbio, Beijing, China), was added to continue the culture for 4 h, 100 μL of the supernatant was discarded, 150 μL of the triplet solution was added to each well, and the mixture was shaken and dissolved for 10 mins. The optical density (OD) value at the wavelength of 570 nm was measured by a microplate reader.

The stimulation index (SI) was calculated: SI = stimulation hole OD value / control hole OD value.

### Determination of serum hemolysin

Sheep red blood cells (SRBC) (Solarbio, Beijing, China) were prepared into 2% (v/v) cell suspensions with normal saline, and on the 7th day of intragastric administration, all groups were intraperitoneally injected with 0.2 mL for sensitization. After immunization for 4 days, blood was taken from the iliac vein in the orbit and centrifuged in a centrifuge tube. After centrifugation at 4000 r·min^− 1^ for 10 mins, the serum was separated and collected, and the serum was diluted 100-fold with physiological saline. The diluted serum 1 mL, 10% SRBC 0.5 mL, and 1 mL 10% complement (Solarbio, Beijing, China) were sequentially added to the test tube. The same volume of physiological saline was used as a blank control, shaken and placed in a 37 °C water bath for 30 min, transferred to an ice bath to terminate the reaction, centrifuged at 2000 r·min^− 1^ for 10 mins, the supernatant was taken, each set with 3 parallel wells, the OD value was measured using a microplate reader (540 nm) [17].

### The phagocytic index α assay using carbon clearance test

On the 11th day, mice in each group were killed after weighed and the phagocytic index α and the phagocytic rate κ of each group have been determined. After the last administration, each group of mice was injected with a dose of 0.1 mL/10 g of Indian ink diluted 4 times with physiological saline through the tail vein, and immediately after the injection, 20 μL of blood was taken at 2 and 10 mins after the ink was injected, and added to 2 mL of 0.1% Na_2_CO_3_ solution, shake well, and use Na_2_CO_3_ solution as blank control to measure the OD at 600 nm, and set up 3 parallel holes. The mice were sacrificed and the liver and spleen were taken and weighed. The phagocytic index α [18] is calculated as follows. α = κ^1/3^ × body weight / (liver weight + spleen weight) where κ = (lgOD_1_-lgOD_2_) / (T_2_-T_1_).

### NK cell activity assay

Target cells (Yac-1 cells, Shanghai Institutes for Biological Sciences, Shanghai, China): Target cells were subcultured 24 h before the experiment. The cells were washed three times with Hank’s solution and adjusted to a cell concentration of 4 × 10^5^ cells/mL with RPMI 1640 complete medium (Thermo Fisher Scientific, Waltham, MA, USA) for use.

Preparation of spleen cell suspension (effector cells): The spleen of the mice was isolated under aseptic conditions, placed in a dish containing an appropriate amount of sterile Hank’s solution, and the fat and connective tissue were removed, ground in a mortar, filtered through a 200 mesh screen, and the filtrate was collected and centrifuged at 1000 r/min for 10 mins, the supernatant was discarded. Add 3 times volume of red blood cell lysate, lyse on ice for 10 mins, centrifuge at 4 °C, discard the supernatant, wash the cells twice with Hank’s solution, resuspend with 0.5 mL of complete medium, count live cells, adjust the number of cells to 2 × 10^7^ cells/mL. Add 100 μL each of effector cell fluid and target cell fluid to a 96-well plate, and set the target cell natural release well (100 μL target cell + 100 μL complete medium) and the maximum release well (100 μL target cell + 100 μL of 1% NP-40 solution (Solarbio, Beijing, China), each set of 3 parallel wells, incubated for 48 h in a 37 °C, 5% CO_2_ incubator.

The supernatant of each well was aspirated and the lactate dehydrogenase (LDH) activity was determined by the LDH kit (Jiancheng, Nanjing, China) and determined OD value at 450 nm.

NK cell activity (%) = (measured well OD - natural release pore OD) / (maximum release pore OD - natural release pore OD) × 100%.

### Blood count tests

Before the animal was sacrificed, blood was taken from the iliac vein in the eyelid. EDTA-K_2_ was added to the test tube as an anticoagulant reagent, and the blood count tests were measured by an automatic blood cell analyzer (Sysmex, Japan).

### Effect of NPPN combined with CTX on tumor inhibition rate in the H22 tumor-bearing mice

#### Grouping and administration of mice

Kunming SPF mice were randomly divided into 9 groups, 10 in each group, followed by (1) control group, (2) saline group, (3) CTX group (25 mg·kg^− 1^), (4) Low dose NPPN group (93 mg·kg^− 1^), (5) medium dose NPPN group (188 mg·kg^− 1^), (6) high dose NPPN group (375 mg·kg^− 1^), (7) NPPN low dose (93 mg·kg^− 1^) + CTX (25 mg·kg^− 1^) group, (8) NPPN medium dose (188 mg·kg^− 1^) + CTX (25 mg·kg^− 1^) group, (9) NPPN high dose (375 mg·kg^− 1^) + CTX (25 mg·kg^− 1^) group, (1), (2), (3) group were orally administered 0.2 mL of normal saline; (4) ~ (9) group were orally administered NPPN; continuous oral administration for 19 days.

#### Inoculation of H22 hepatoma cells and intraperitoneal injection of CTX

On the morning of the 6th day of administration, mice were given H22 hepatoma cells (National Infrastructure of Cell Line Resource, Beijing, China) for 7 days in the abdominal cavity of mice, and the mouse abdominal cavity was swabbed with 75% ethanol, and the sodium chloride injection in the syringe was pushed to mouse abdominal cavity, then take the mouse ascites, repeat several operations, get H22 liver cancer cell suspension, take 10 μL of cell suspension in a clean EP tube, add 1990 μL saline, mix and count. The concentration of the saline cells was adjusted to 2.5 × 10^7^ cells/mL, and the mice in the (2) ~ (9) group were inoculated with 0.2 mL of tumor cell suspension. After inoculation, mice in groups (3), (7), (8) and (9) were intraperitoneally injected with cyclophosphamide drug solution for 14 consecutive days.

#### Determination of tumor inhibition rate in the H22 tumor-bearing mice

On the 14th day after inoculation, after the last administration, the mice fasted for 12 h, and the mice were sacrificed by cervical dislocation. The tumor tissues were removed and weighed. The tumor inhibition rate (%) = (1 - treatment group mean tumor weight / saline group mean tumor weight) × 100%.

### Statistical methods

The LSD analysis of variance (ANOVA) was used for the significance test. Data were all presented as EMBED Eq. 3 $\overline{x}\pm s$; one-way ANOVA and comparison among groups were performed using the SPSS 17.0 statistical software and a probability of less than 0.05 (*p* < 0.05) was considered statistically significant.

## Results

### Extraction of NPPN from CPPN

**Fig. 1:**
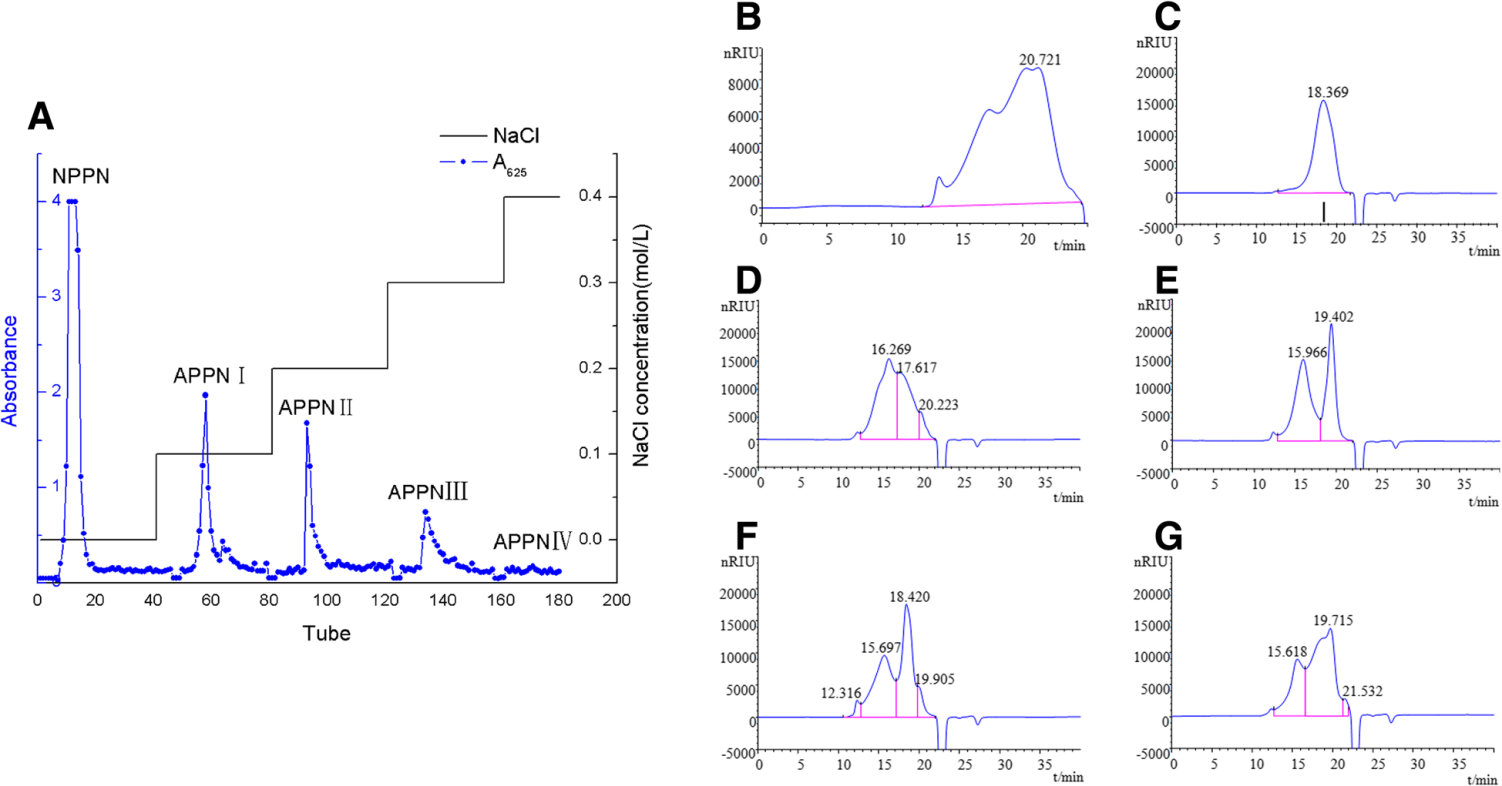
**a** Elution curve of CPPN via DEAE Sepharose Fast Flow anion exchange chromatography column. **b** High performance gel permeation chromatogram of CPPN. **c** High performance gel permeation chromatogram of NPPN. **d** High performance gel permeation chromatogram of APPNI. **e** High performance gel permeation chromatogram of APPNII. **f** High performance gel permeation chromatogram of APPNIII. **g** High performance gel permeation chromatogram of APPNIV

In our previous report, we were able to isolate CPPN from the herb residue of *Panax notoginseng* with the content of polysaccharides is 56.2% and the application of CPPN prolonged the lifespan of H22 tumor-bearing mice by boosting the immune system [13]. To further validate that the polysaccharide portion of the CPPN played a major role in the boosting of the immune system, CPPN was further purified by an anion-exchange chromatography column of DEAE Sepharose Fast Flow to enrich the polysaccharide portion. The NPPN was eluted with water and four APPN fractions (APPN I, APPN II, APPN III, APPN IV) were eluted with and 0.1, 0.2, 0.3, 0.4 mol·L^− 1^ sodium chloride solutions respectively (Fig. 1). After the dialysis and freeze-dried, the yields of the NPPN, APPN I, APPN II, APPN III, and APPN IV were 30.51, 29.56, 14.72, 7.43, 2.54% respectively. The Mw, Mn and dispersity of each fraction and the contents of polysaccharides were measured. The results are shown in Table 1. The contents of polysaccharides of NPPN was the highest, which increased to 88.4% from 56% of CPPN (Table 1). The HPGPC analysis revealed that NPPN was the purest extraction from *Panax notoginseng* with a single peak (Fig. 1c) compared to other extractions (Fig. 1b, d, e, f, and g). Thus, NPPN was selected in the future experiment combined with CTX treatment.

**Table 1:** The molecular weights and the contents of polysaccharides from *Panax notoginseng*

Sample	Mn	Mw	D (Mw/Mn)	Content (%)
CPPN	9207	97,660	10.61	56.2
NPPN	6906	18,100	2.62	88.4
APPN I	7708	70,470	9.142	76.1
APPN II	15,190	105,600	6.953	48.3
APPN III	9431	92,330	9.79	36.2
APPN IV	10,060	90,170	8.967	32.4

### Administration of NPPN with CTX ameliorated the immunosuppression caused by CTX

CTX is a well-known immunosuppressant and chemotherapy drug for cancer treatment, which also causes severe immunosuppression and myelosuppression. Using the organ indexes of the spleen, and thymus, the two major immune organs of a mouse, we evaluate the overall impaction of NPPN on the immune status in CTX treated mice, compared to the normal group, both spleen and thymus indexes of the CTX-treated group were reduced significantly (*P* < 0.01), which indicated the immunosuppression resulted from CTX treatment (Table 2). Compared to the CTX-treated group, the high-dose NPPN plus cyclophosphamide group increase thymus indexes (*P* < 0.05).

**Table 2:** Effect of NPPN on immune function in mice with immunosuppression caused by CTX ($\overline{x}\pm s$, *n* = 10)

Group	Dosage (mg·kg^−1^)	Thymus index	Spleen index	Stimulation index	Absorbance value of serum hemolysin	NK cell activity (%)	κ	α
Normal	–	3.80 ± 0.80**	3.05 ± 0.68**	1.07 ± 0.05**	0.59 ± 0.01**	62.16 ± 4.59**	0.023 ± 0.007*	5.12 ± 0.37**
CTX	80	0.68 ± 0.08	1.17 ± 0.13	0.91 ± 0.05	0.36 ± 0.04	25.42 ± 8.33	0.017 ± 0.004	4.69 ± 0.25
NPPN-L + CTX	93 + 80	0.76 ± 0.09	1.33 ± 0.29	0.96 ± 0.04**	0.55 ± 0.05**	35.56 ± 6.47**	0.024 ± 0.006*	4.94 ± 0.47
NPPN-M + CTX	188 + 80	0.86 ± 0.12	1.22 ± 0.27	0.97 ± 0.05**	0.57 ± 0.03**	29.07 ± 5.27	0.022 ± 0.004	4.77 ± 0.40
NPPN-H + CTX	375 + 80	0.98 ± 0.06*	1.29 ± 0.17	0.94 ± 0.01	0.57 ± 0.03**	35.13 ± 5.93**	0.025 ± 0.005**	5.10 ± 0.28**

Notes: * *P* < 0.05, ** *P* < 0.01 vs. CTX

**Fig. 2:**
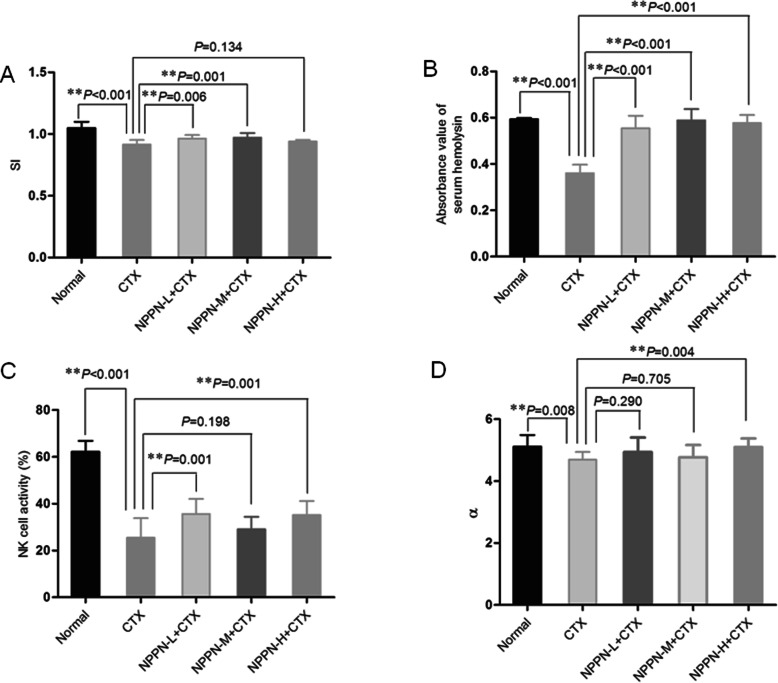
Effect of NPPN on immune function in mice with immunosuppression caused by CTX. Notes: **a** Stimulation index. **b** Serum hemolysin antibody content. **c** NK cell activity. **d** Phagocytic index

We further evaluated the effect of NPPN application on cellular and humoral immunity as the indicators of the strength of the immune system including the proliferation of T lymphocytes and the measurement of the serum hemolysin. When T lymphocytes are stimulated by Concanavalin, a proliferative response occurs in the mother cells. Mitochondrial hydrolase in living cells, especially in proliferating cells, can decompose MTT into blue-purple crystals, and its optical density value can reflect the proliferation of T lymphocytes. The result of the measurement of the proliferation of T lymphocytes was shown in Table 2 and Fig. 2a. Compared with the normal group, the proliferation of T lymphocytes in the CTX group was significantly decreased than that in the normal group (*P* < 0.01), indicating that the activity of the proliferation of T lymphocytes was suppressed. When NPPN was applied together with CTX, though the highest dosage of NPPN was combined with CTX increased the proliferation of T lymphocytes of mice without significance, both lowest and medium dosages significantly increase the proliferation of T lymphocytes compared to the CTX-treated group(*P* < 0.01) (Fig. 2a).

The mechanism of measuring the serum hemolysin content of mice is that sheep red blood cells enter the body and stimulate the body to produce specific antibody hemolysin, which is an anti-sheep red blood cell antibody. The antigen-antibody binds in vitro to form an antigen-antibody complex, exposing the C1q binding points of complement, activating complement, and causing sheep red blood cells to lyse. By examining the amount of antibodies produced by the body, the body’s humoral immune function status is reflected. The result of the measurement of the serum hemolysin was shown in Table 2 and Fig. 2b. Compared with the normal group, the serum hemolysin antibody content in the CTX group was significantly decreased (*P* < 0.01), indicating that the production of serum hemolysin antibodies was suppressed. When NPPN was applied together with CTX, all serum hemolysin contents of the low, medium and high dose groups were significantly higher than that of the CTX group (*P* < 0.01). In addition, all serum hemolysin contents of the low, medium and high dose groups had increased to the same level as the normal group, which indicated a full recovery of the activity of antibody hemolysin antibody under the NPPN and CTX combined treatment.

We further evaluated the effect of NPPN application on specific cell activities as the indicators of the strength of the immune system including NK cell activity and phagocytic capability of macrophage. As shown in Table 2 and Fig. 2c, compared with the normal group, the application of CTX strongly deceased the NK cell activity (*P* < 0.01), which indicated the immunosuppression of the CTX treated mice. When NPPN was applied to mice with CTX at different dosages, the NK cell activity of the low and high dose groups was significantly higher than that of the CTX group (*P* < 0.01). The result suggests NPPN increased the NK cell activity.

**Fig. 3:**
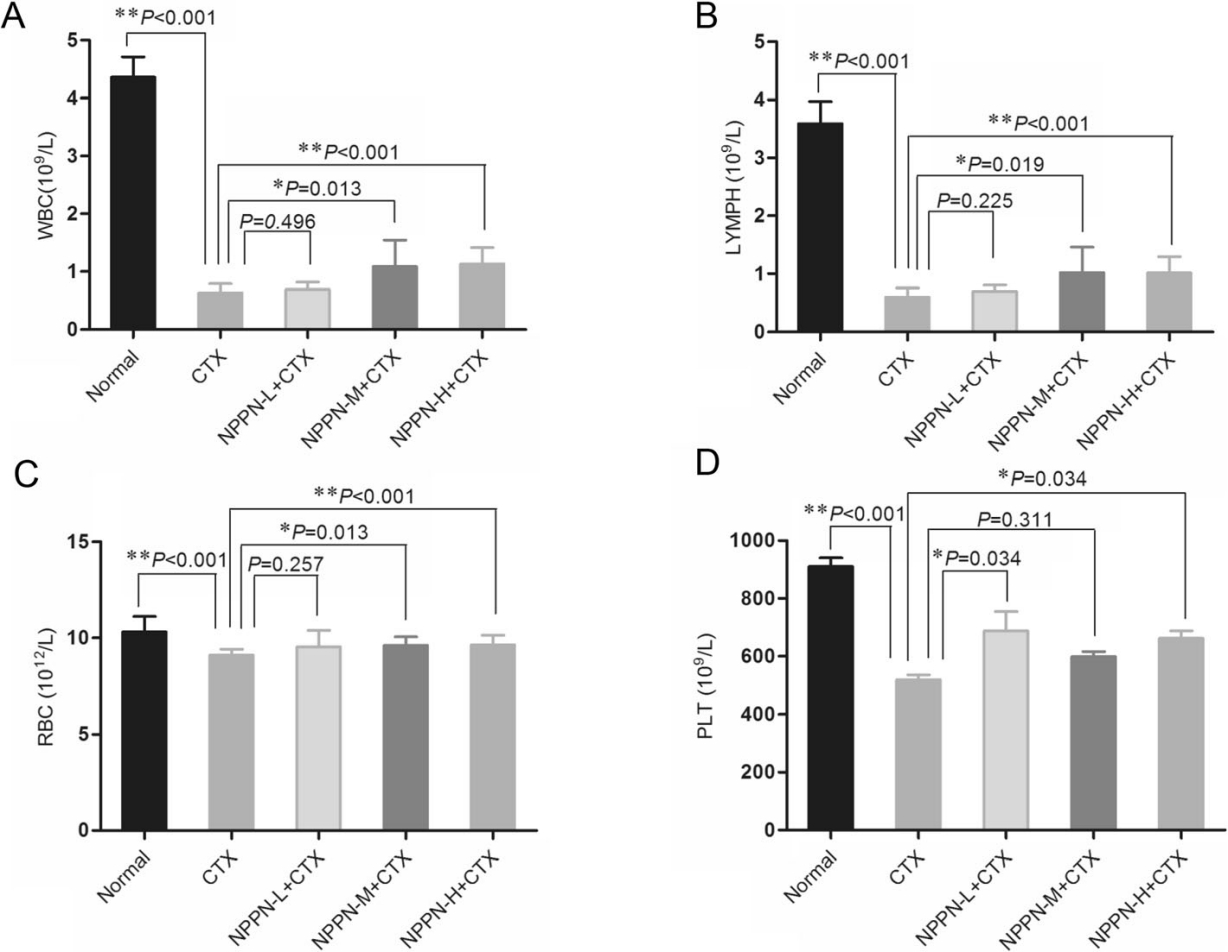
Effect of NPPN on hematological parameters in mice with myelosuppression caused by CTX. Notes: **a** WBC. **b** LYMPH. **c** RBC. **d** PLT

Phagocytosis of macrophages is also a crucial indicator of immune function. We perform the carbon clearance test to evaluate the phagocytic capability of macrophages. The results of the carbon clearance test are shown in Table 2 and Fig. 3d. The phagocytic index α of the CTX group were significantly lower than those of the control group (*P* < 0.05), indicating the immunosuppression established by CTX. Though the lowest and middle dosage of NPPN was combined with CTX increased, the high dosages significantly increase the phagocytic indexes compared to the CTX-treated group (*P* < 0.01) (Fig. 2d). This result indicates the combination of NPPN with CTX improved the macrophage activity suppressed by CTX.

### NPPN combined with CTX improved myelosuppression caused by CTX treatment

After the evaluation of NPPN improved the immunosuppression caused by CTX, we investigated the effect of NPPN on the amelioration of myelosuppression caused by CTX, when NPPN was applied together with CTX using blood cell analysis. Four blood cell count rests were determined by an automatic blood cell analyzer. The results of blood count tests are shown in Table 3. 4 blood cell count results in the CTX group were significantly lower than those in the normal group (*P* < 0.05 or *P* < 0.01) including white blood cells, lymphocyte numbers, red blood cell and platelet.

**Table 3:** Effect of NPPN on hematological parameters in mice with immunosuppression ($\overline{x}\pm s$, *n* = 10)

Group	Dosage (mg·kg^− 1^)	WBC(10^9^/L)	LYMPH(10^9^/L)	RBC(10^12^/L)	PLT(10^9^/L)
Normal	–	4.35 ± 0.81**	3.57 ± 0.39**	10.32 ± 0.80**	812.2 ± 89.1**
CTX	80	0.62 ± 0.12	0.60 ± 0.17	9.11 ± 0.34	520.70 ± 52.17
NPPN-L + CTX	93 + 80	0.69 ± 0.12	0.71 ± 0.25	9.53 ± 0.87	687.50 ± 211.04*
NPPN-M + CTX	188 + 80	1.08 ± 0.35*	1.02 ± 0.44**	9.62 ± 0.44	598.70 ± 55.26
NPPN-H + CTX	375 + 80	1.10 ± 0.31**	1.02 ± 0.28**	9.67 ± 0.47*	661.80 ± 83.47*

Notes: * *P* < 0.05, ** *P* < 0.01 vs. CTX

This result indicates that the CTX treatment resulted in myelosuppression status in mice (Table 3 and Fig. 3). When NPPN was applied together with CTX, four blood cell count indicators showed significant increases up to the levels of the CTX group including the white blood cell, lymphocyte numbers, red blood cell, platelet (*P* < 0.01 or *P* < 0.05). The white blood cell (Fig. 3a) and lymphocyte numbers (Fig. 3b) in the medium and high dose groups of NPPN were higher than that of the CTX group (*P* < 0.01 or *P* < 0.05); the red blood cell (Fig. 3c) in the high dose groups of NPPN were higher than that of the CTX group (*P* < 0.05); the platelet counts (Fig. 3d) of the low and high dose groups of NPPN were significantly higher than those of the CTX group (*P* < 0.05).

### NPPN combined with CTX enhanced tumor inhibition effect of CTX on H22 tumor-bearing mice

**Fig. 4:**
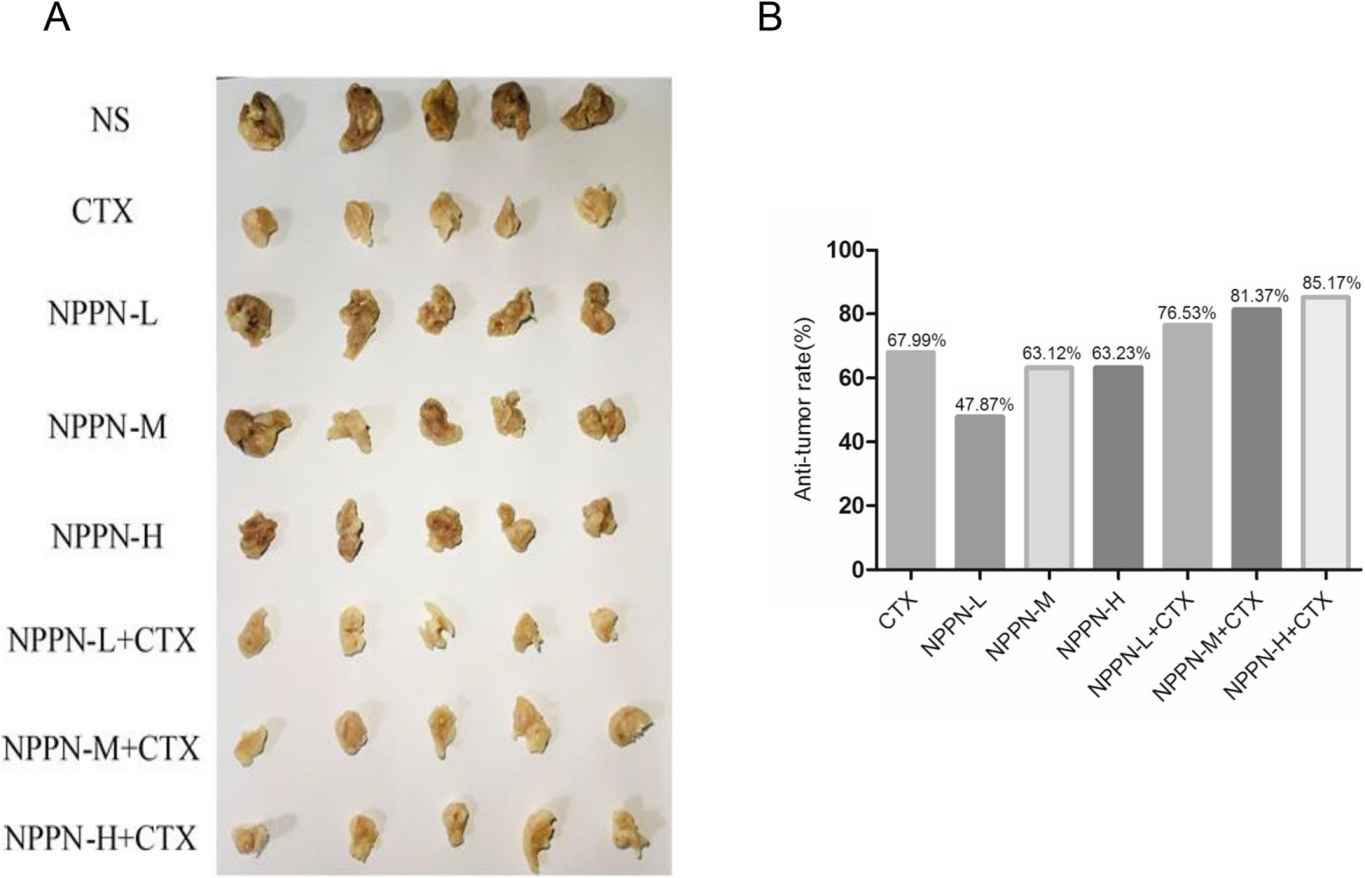
Effects of NPPN combined with CTX on inhibition rate in H22 tumor-bearing mice. Notes: **a** Tumor images of each experimental group of tumor-bearing mice. **b** The result of anti-tumor rate

The previous results indicated NPPN combined with CTX as an immunostimulator ameliorated the immunosuppression and myelosuppression resulted from the administration of CTX alone. We investigated the antitumor activity of the combination treatment with NPPN and CTX on H22 liver tumor-bearing mice. The results are shown in Table 4 and Fig. 4. When CTX was applied to the H22 tumor-bearing mice alone, the tumor inhibition rate reached 67.99% compared with the normal group treated with saline. The application of NPPN alone showed 47.87, 63.12 and 63.23% tumor inhibition rates at the low, medium and high NPPN dosage compared with the normal group treated with saline, which is consistent with our previous report that CPPN with only 56% of polysaccharide contents prolonged the lifespan of the H22 tumor-bearing mice. This result indicates the polysaccharide portion of CPPN plays a major role in its antitumor activity against HCC [14]. When NPPN combined with CTX, both medium dosage and high dosage of NPPN combined with CTX groups showed significant increases in tumor inhibition rates compared with the CTX group alone (*P* < 0.05).

**Table 4:** Effects of NPPN combined with CTX on inhibition rate in H22 tumor-bearing mice (EMBED Eq. 3 $\overline{x}\pm s$, *n* = 5)

Group	Dosage (mg·kg^− 1^)	Tumor weight	Anti-tumor rate (%)
Normal	–	–	–
NS	–	1.36 ± 0.10**	–
CTX	25	0.44 ± 0.12^##^	67.99
NPPN-L	93	0.71 ± 0.25^##^ *	47.87
NPPN-L + CTX	93 + 25	0.32 ± 0.04##	76.53
NPPN-M	188	0.50 ± 0.20^##^	63.12
NPPN-M + CTX	188 + 25	0.25 ± 0.05## *	81.37
NPPN-H	375	0.50 ± 0.17^##^	63.23
NPPN-H + CTX	375 + 25	0.20 ± 0.05^##^ *	85.17

Notes: ^#^ *P* < 0.05, ^##^ *P* < 0.01 vs. NS; * *P* < 0.05, ** *P* < 0.01 vs. CTX

## Discussions

Our previous studies demonstrated that CPPN administration could effectively prolong the lifespan of tumor-bearing mice via boosting the host immune system as well as weak cytotoxicity against HCC. CPPN may have a potential application for the treatment of hepatocellular carcinoma [14]**.** In order to explore the pharmacodynamics substance basis of Panax notoginseng polysaccharide, the CPPN was further purified into NPPN and APPN. The effects of NPPN and three APPN on the proliferation of seven tumor cells in vitro were investigated in the previous experiments. The seven tumor cells were: H22 mouse hepatoma cells, S180 mouse ascites tumor cells, YAC-1 lymphocytes. Tumor cells, MCF-7 human breast cancer cells, 4 T1 mouse breast cancer cells, B16 mouse melanoma cells and CT26.WT mouse colon cancer cells, it was found that only NPPN inhibited the growth of each cell line. The pre-experiment also examined the effects of NPPN and three kinds of APPN on the proliferation of human periodontal ligament stem cells and mouse osteoblasts in vitro*,* the results showed that NPPN and APPN I on human periodontal Membrane stem cells have the effect of promoting proliferation. NPPN, APPN I, APPN II and APPN III can promote the proliferation of mouse osteoblasts. Therefore, this experiment used NPPN as experimental samples.

In this study, a CTX-induced immunosuppression combined with a myelosuppressive mouse model was established by intraperitoneal injection of CTX at a dose of 80 mg/kg/d for 5 days. The NPPN was continuously administered to the stomach for 10 days, and the mice were sacrificed. The peripheral blood was measured by an automatic blood cell analyzer. Four blood cell count results were determined by blood cell analyzer and the 4 blood cell count results were higher than that of the CTX group (*P* < 0.01 or *P* < 0.05). The phagocytosis ability and serum hemolysin level determined by peripheral blood were not different between the NPPN group and the normal group. The NK cell activity and lymphocyte proliferative response measured by spleen were significantly lower in the NPPN group than in the normal group (*P* < 0.01), suggesting that the main target of the NPPN in CTX-induced immunosuppression combined with myelosuppressive mouse model is in the blood system. Cancer chemotherapy drugs are aimed at actively growing cells. In addition to malignant tumor cells, cells in bone marrow hematopoietic stem cells, digestive tract mucosa, skin and its appendages, endometrium, and ovary are also rapidly renewed. This is the histological basis for the corresponding adverse effects of chemotherapy drugs. It can be considered that almost all chemotherapeutic drugs have myelosuppressive effects, the difference is only in degree. CTX is an alkylating agent antitumor drug and a cytotoxic immunosuppressive drug. It is a broad-spectrum anti-tumor drug, effective for leukemia and solid tumors, and one of the most potent and widely used drugs among various immunosuppressants currently in use. Due to its good clinical efficacy, the application range is increasingly wide. CTX enters the body and is hydrolyzed by excess phosphoramidase or phosphatase present in the liver or tumor to act as activated phosphoramide mustard. It cross-links with DNA, inhibits DNA synthesis, and interferes with RNA function. It is a cell cycle non-specific drug. Compared with other cytotoxic drugs, immunosuppressive effects are strong and long-lasting. CTX has a greater toxic side effect and myelosuppression is the most common toxicity. In conclusion, the NPPN has a repairing effect on hematopoietic function and immunosuppression induced by cyclophosphamide in mice, and it is speculated that the target is bone marrow hematopoietic stem cells, which has a repairing effect on DNA damage of bone marrow hematopoietic stem cells.

Based on NPPN, it can repair the hematopoietic function and immunosuppression to a certain degree induced by CTX in mice. We treated NPPN with CTX in H22 tumor-bearing mice, and found that the tumor weight was significantly lower in the NPPN medium and high-dose + CTX group compared with the CTX group (*P* < 0.05). Therefore, the combination of chemotherapeutic drugs and, NPPN which are cytotoxic to various tumor cells and has a repairing effect on hematopoietic function and immunosuppression caused by chemotherapy after bone marrow suppression in mice, will provide new possibilities for the treatment of tumors.

Nitrogen mustards (NMs) form cyclic aminium ions (aziridinium rings) by intramolecular displacement of the chloride by the amine nitrogen. This aziridinium group then alkylates DNA once it is attacked by the N-7 nucleophilic center on the guanine base. A second attack after the displacement of the second chlorine forms the second alkylation step that results in the formation of interstrand cross-links (ICLs) as it was shown in the early 1960s. At that time it was proposed that the ICLs were formed between N-7 atom of guanine residue in a 5′-d (GC) sequence [19]. Later it was clearly demonstrated that NMs form a 1, 3 ICL in the 5′-d (GNC) sequence [20–23].

The strong cytotoxic effect caused by the formation of ICLs is what makes NMs an effective chemotherapeutic agent. Other compounds used in cancer chemotherapy that have the ability to form ICLs are cisplatin, mitomycin C, carmustine, and psoralen [24]. These kinds of lesions are effective at forcing the cell to undergo apoptosis via p53, a protein that scans the genome for defects. Note that the alkylating damage itself is not cytotoxic and does not directly cause cell death [25].

NPPN has a protective effect on immune organs such as bone marrow, spleen and thymus in immunosuppressed mice induced by cyclophosphamide. The serum hemolysin level and macrophage phagocytosis index measured from peripheral blood showed no statistically significant difference between the high-dose NPPN group and the normal group, while the NK cell activity and T-lymphocyte proliferation index measured from the spleen showed a significant difference compared with the normal group. It suggests that NPPN has a stronger protective effect on peripheral blood than the spleen, and peripheral blood comes from bone marrow, suggesting that NPPN has a protective effect on bone marrow. Immunosuppressive cyclophosphamide targets are proliferative cells, while bone marrow stem cells are proliferative cells. The protective effect of NPPN on bone marrow stem cells and bone marrow is underway in our research group.

## Conclusion

CPPN was separated and purified by DEAE Sepharose Fast Flow anion exchange column. The eluted part of H_2_O was dialyzed and freeze-dried to obtain NPPN. The content of polysaccharide in the NPPN was determined by the anthrone-sulfuric acid method to be 88.4%, and the yield was 28.9%. The weight-average molecular weight of the NPPN measured by high-performance gel chromatography was 18,100 Da, and the pH value of the NPPN solution was 7.35.

NPPN can increase the thymus index, cellular immunity, humoral immunity, and bone marrow hematopoietic function of immunosuppressive and bone marrow-suppressed mice induced by the chemotherapy drug CTX.

NPPN were used to treat H22 tumor-bearing mice. Compared with the saline group, NPPN low, medium and high dose groups had higher tumor inhibition rates and significant differences (*P* < 0.01), the tumor inhibition rate of the medium and high dose group of NPPN was not different from that of the cyclophosphamide group. Compared with the cyclophosphamide group, the tumor inhibition rate of the medium and high doses of NPPN and cyclophosphamide combined increased the tumor inhibition rate and had significant differences (*P* < 0.05).

## Data Availability

All data generated or analysed during this study are included in this published article.

## Abbreviations

CPPN: Crude polysaccharide from *Panax notoginseng*
NPPN: Neutral polysaccharide from *Panax notoginseng*
APPN: Acid polysaccharide from *Panax notoginseng*
CTX: Cyclophosphamide
HCC: Hepatocellular carcinoma
Mw: Average molecular weight
Mn: Number average molecular weight
HPGPC: High performance gel permeation chromatography
MTT: 3-(4, 5-dimethyl-2-thiazolyl)-2, 5-diphenyl-2-H-tetrazolium bromide
OD: Optical density
SI: Stimulation index
SRBC: Sheep red blood cells
LDH: Lactate dehydrogenase
WBC: White blood cell
LYMPH: Lymphocyte
RBC: Red blood cell
PLT: Platelet
